# Supplementary material for: Transforming Growth Factor-beta signaling in αβ thymocytes promotes negative selection
Source: Nat Commun. 2019 Dec 19;10:5690. doi: 10.1038/s41467-019-13456-z (PMC6923358; doi:10.1038/s41467-019-13456-z)
Supplement: Supplementary file 1 — Supplementary Information [file 41467_2019_13456_MOESM1_ESM.pdf]

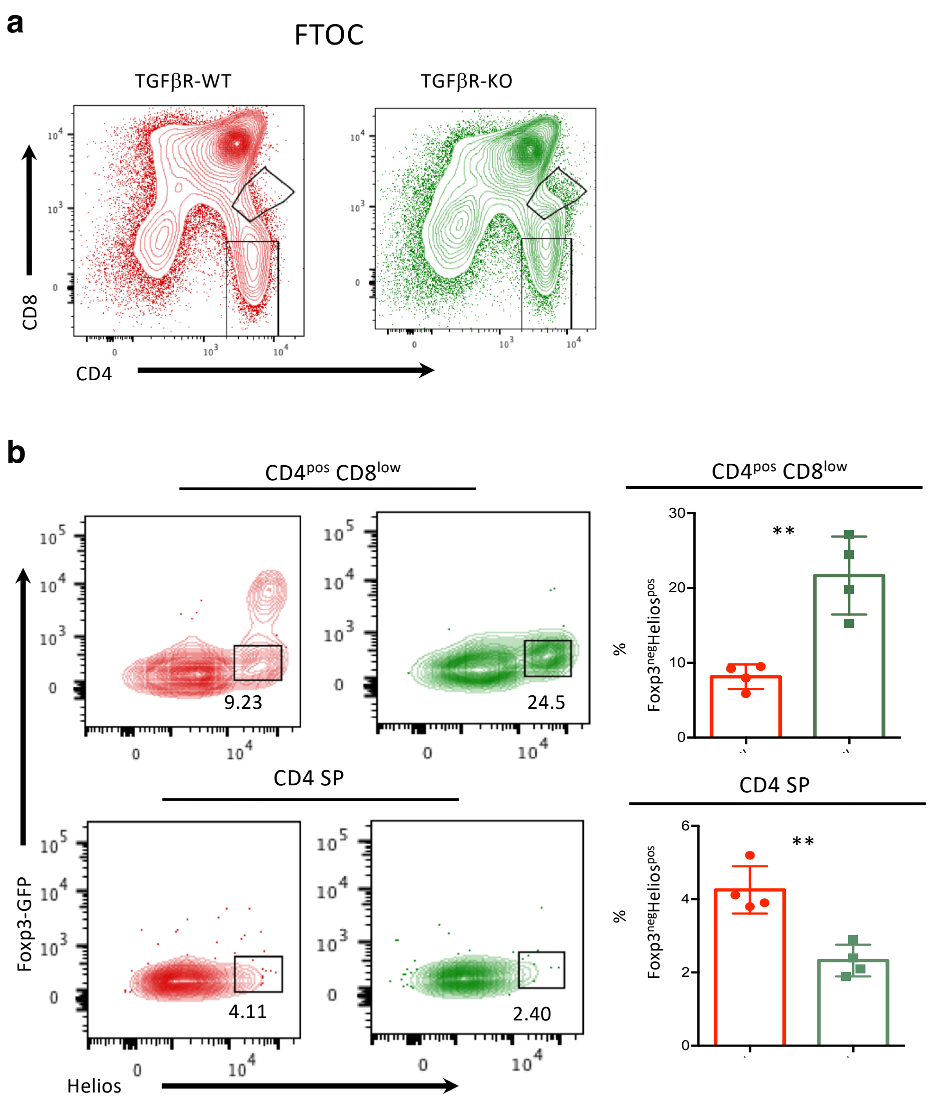


**Supplementary Figure 1. Exacerbated presence of Foxp3$^{neg}$ Heliospos CD4$^{pos}$ CD8$^{low}$ thymocytes during FTOC derived from TGF-βR-KO mice.**

Flow cytometry analysis of thymus from E17.5 TGFβR-WT mice and TGF-βR-KO mice after 3 days of FTOC. (**a**) Gating strategy for flow cytometry analysis of CD4$^{pos}$ CD8$^{low}$ and CD4SP thymocytes. (**b**) Representative dot plot analysis of the expression of Foxp3 and Helios among the CD4$^{pos}$ CD8$^{low}$ and CD4SP thymocytes. Graphs illustrate the percentage of Foxp3$^{neg}$ Helios$^{pos}$ cells among CD4$^{pos}$ CD8$^{low}$ and CD4SP thymocytes. Data are representative of two independent experiments with 4 mice per groups. (two-tailed Student's t test). ** P<0.01 Error bar, mean ± SEM.

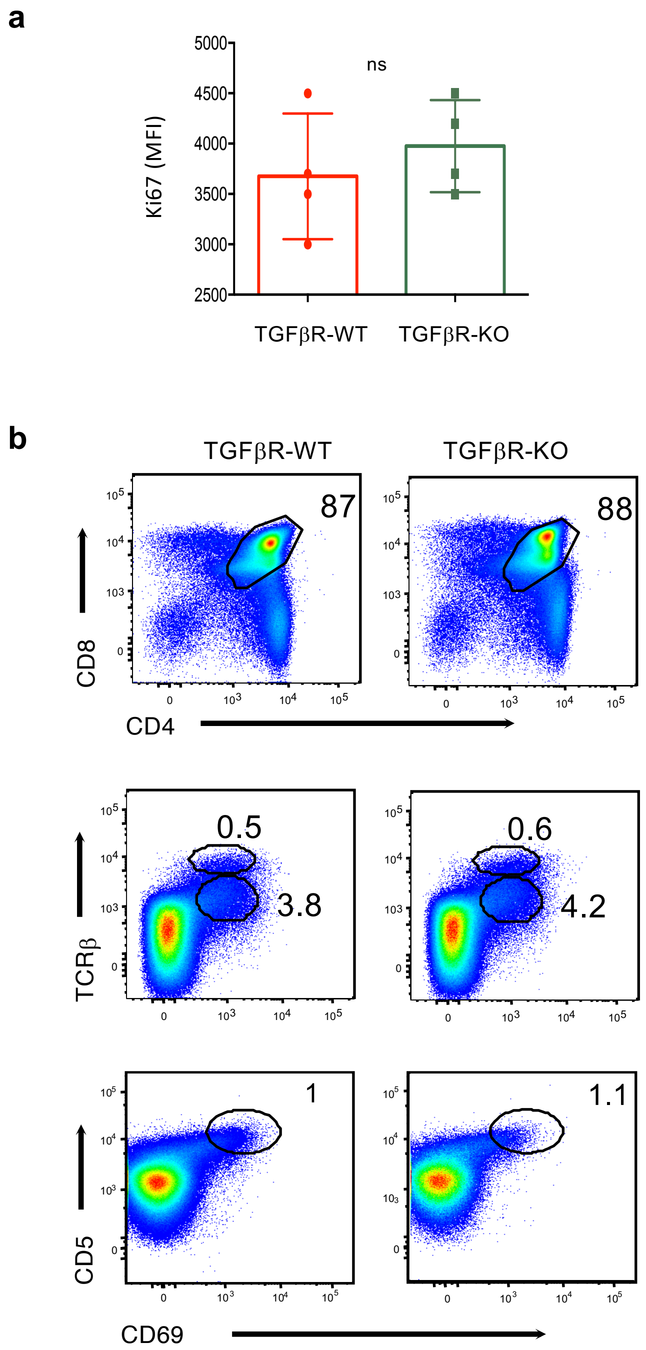


**Supplementary Figure 2. No exacerbated proliferation of Foxp3$^{neg}$ Helios$^{pos}$ CD4$^{pos}$ CD8$^{low}$ thymocytes and positive selection in TGF-βR-KO mice**

**(a)** Foxp3$^{neg}$ Helios$^{pos}$ CD4$^{pos}$ CD8$^{low}$ thymocytes from TGFβR-KO mice and their littermate control (TGFβR-WT) were stained with anti-Ki67 and analyzed by flow cytometry based on gating strategy fig1. Graph illustrates the mean of flow fluorescence MFI. (**b**)**,** Gating strategy for flow cytometry analysis of DP thymocytes from TGF-βR-WT mice and TGF-βR-KO mice. DP cells were analyzed for TCR, CD5 and CD69 markers associated with positive selection. Data are representative of two independent experiments with 4 mice per groups. ns: no statically significant (two-tailed Student's t test). Error bar, mean ± SEM.

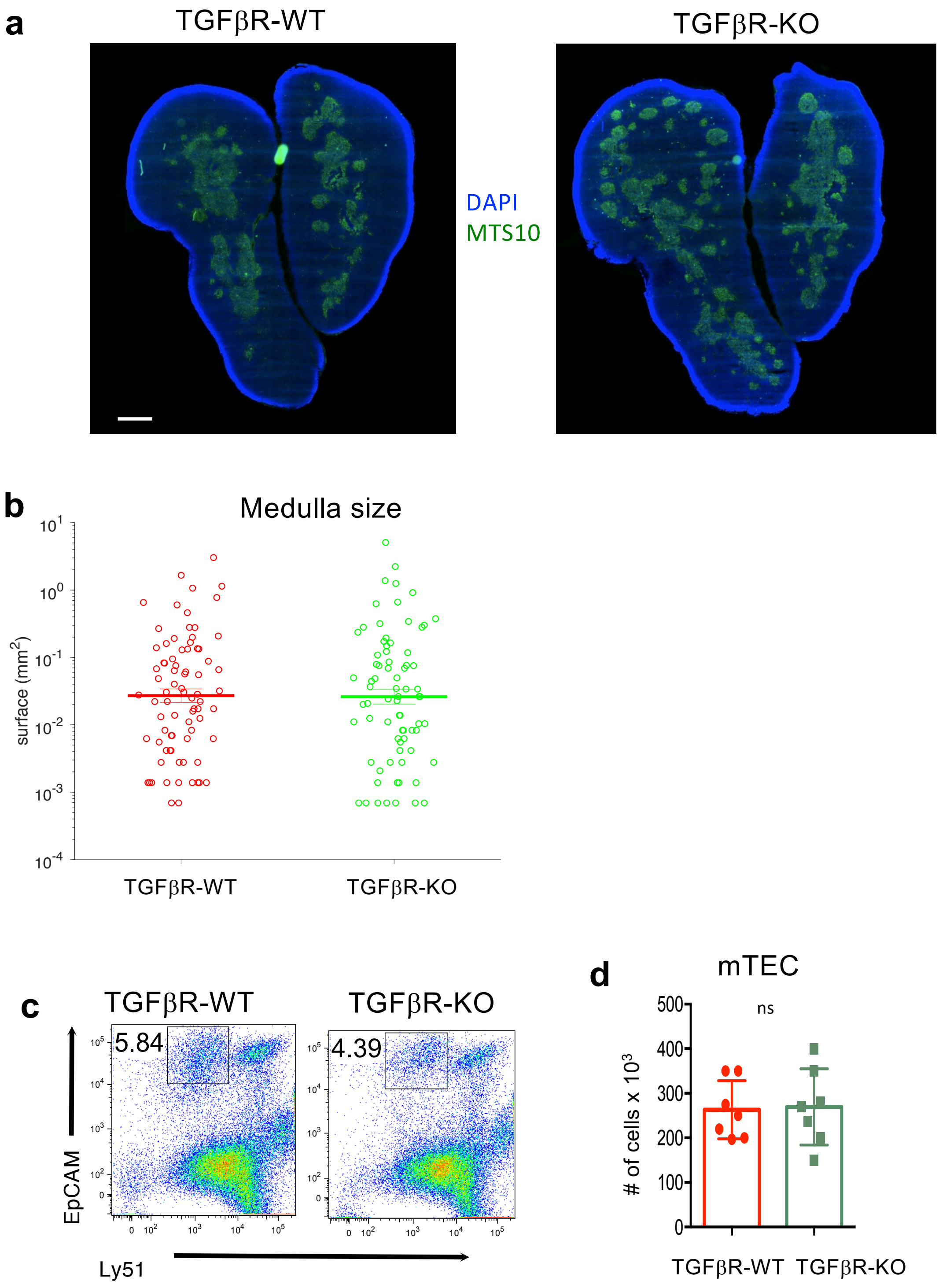


**Supplementary Figure 3. No effect on medulla structure and mTEC apoptosis in TGFβR-KO mice** (**a**) Immunostaining on thymus sections from TGFβR-KO mice and their littermate control (TGFβR-WT). The medulla was stained with MTS10; Scale bar 1mm. (**b**) Graph illustrates the medulla size quantified using Matlab. Statistic analysis were performed using Kolmogorov-Smirnov test for normality and two-tailed Student's t test. (**c**) Gating strategy for mTEC (EpCAM$^{pos}$ Ly51$^{neg}$) flow cytometry analysis on CD45$^{neg}$ thymic cells (**d**) Flow cytometry analysis was performed on mTEC . Graph illustrates the absolute numbers of mTEC in TGFβR-KO mice and their littermate control (TGFβR-WT). Data are representative of 2-4 experiments, with 3-5 mice per group. ns: no statically significant (two-tailed Student's t test). Error bar, mean ± SEM.

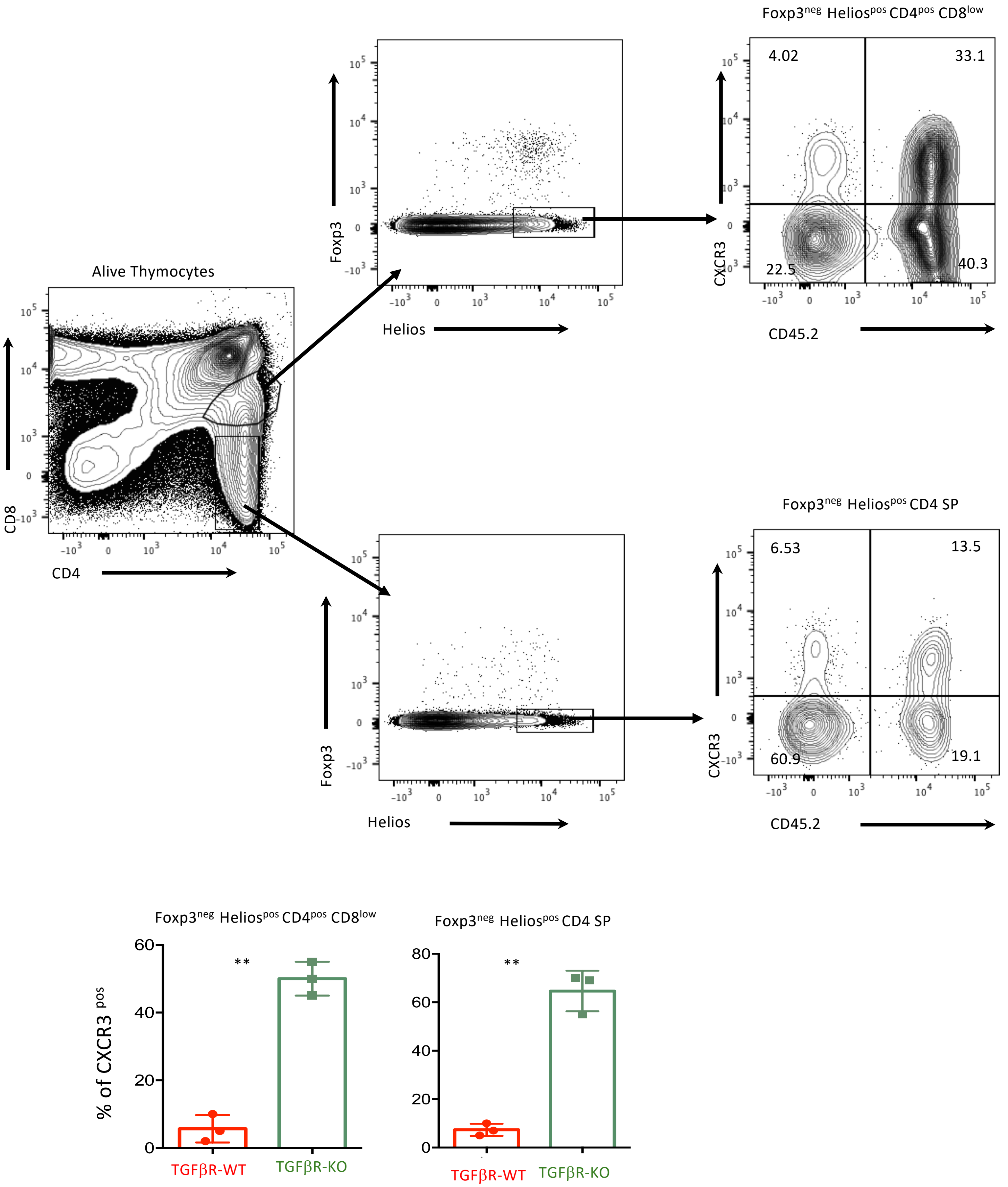


**Supplementary Figure 4. Direct effect of TGF-β signaling on CXCR3 expression**

T cell-depleted BM from TGF-βR-KO mice (CD45.2) were mixed with T cell-depleted BM from congenic TGF-βR-WT(CD45.1) at 1:1 ($10^6$ cells each) and transferred into irradiated Rag2-KO mice. 4.5-5 weeks later, thymuses were analyzed by flow cytometry. Dot plots demonstrate the proportion of Foxp3$^{neg}$ Helios$^{pos}$ CD4$^{pos}$ CD8$^{low}$ thymocytes and Foxp3$^{neg}$ Helios$^{pos}$ CD4 SP thymocytes derived from either TGF-βR-WT BM (CD45.2 $^{neg}$) or TGF-βR-KO BM (CD45.2 $^{pos}$) that express CXCR3 at their surface. Data are representative of 3 animals. Graphs illustrates the percentage of cells from each BM donor. Error bar, mean ± SEM (two-tailed paired Student's t test) ** P<0.01.

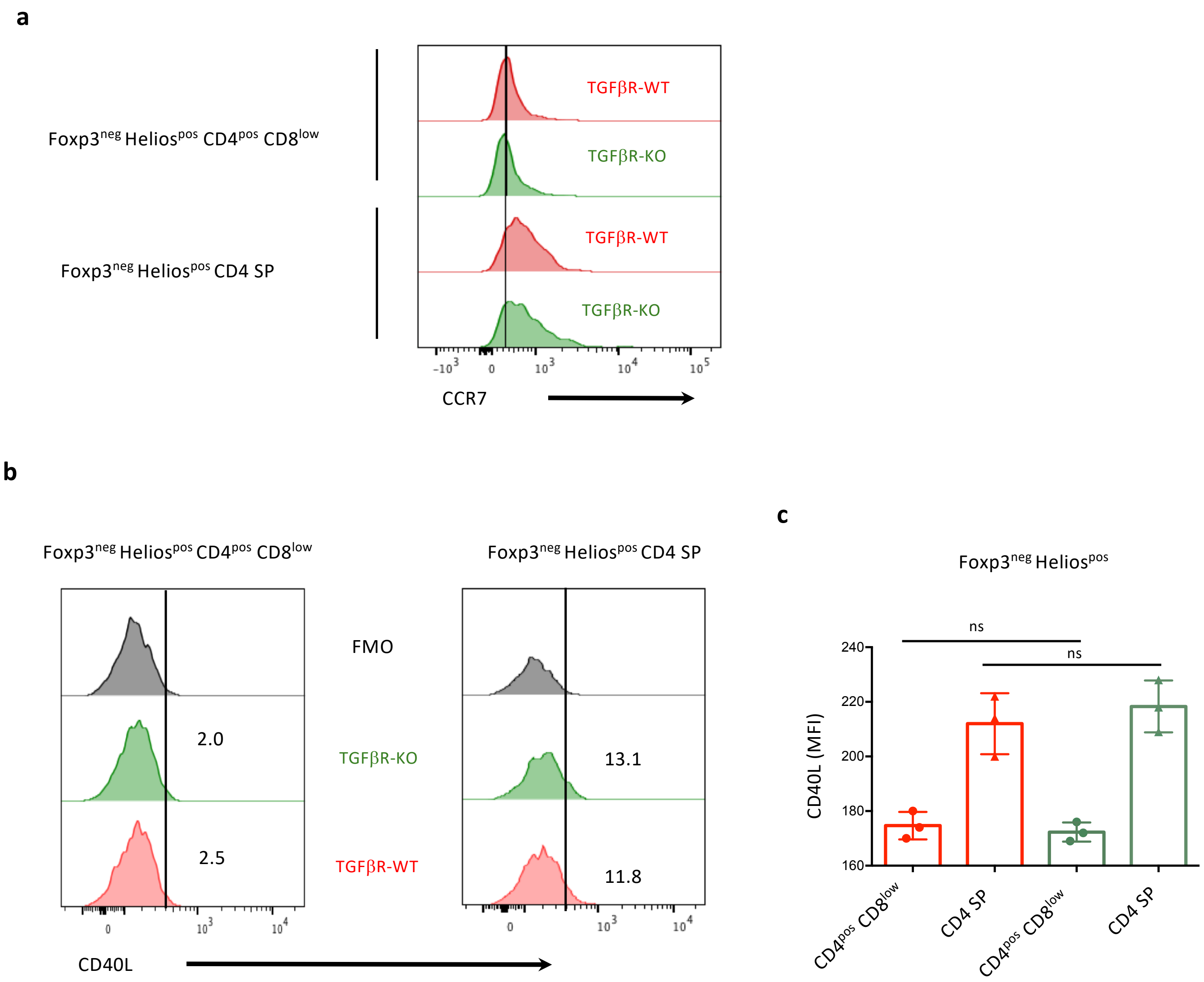


**Supplementary Figure 5. TGF-β signaling does not affect CD40L and CCR7 expression in Foxp3$^{neg}$ Helios$^{pos}$ thymocytes**

(**a-c**) Flow cytometry analysis of the surface expression of CCR7 and CD40L (CD154) in Foxp3$^{neg}$ Helios$^{pos}$ thymocytes from TGF-bR-KO mice and their wild type littermates based on flow cytometry strategy fig1. **c**) Graph illustrates the mean of fluorescence intensity (MFI) of CD40L expression. All the experiments were conducted in 7-10 day-old animals. The data are representative of three animals per groups. ns no statically significant, Error bar, mean ± SEM (two-tailed paired Student's t test).

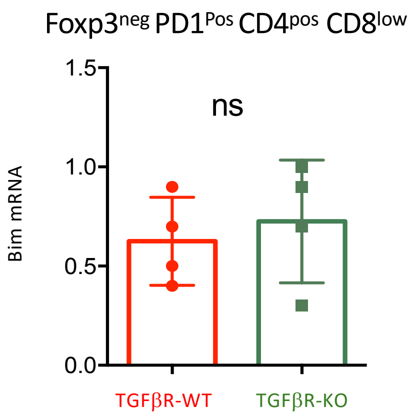


**Supplementary Figure 6. No effect on *Bim* mRNA expression in highly autoreactive TGFβR-KO thymocytes**

FACS sorted Foxp3$^{neg}$ PD1$^{pos}$ CD4$^{pos}$ CD8$^{low}$ thymocytes from TGFβR-WT mice TGFβR-KO mice as illustrated Figure 4c. Histogram illustrates the relative expression of *bim* measured by q-RT-PCR and normalized on *gadph* expression. Data are representative of 2 experiments, with 4 mice per group. ns no statically significant (two-tailed Student's t test) were observed. Error bar, mean ± SEM.
