# Supplementary material for: Transforming Growth Factor-beta signaling in αβ thymocytes promotes negative selection
Source: Nat Commun. 2019 Dec 19;10:5690. doi: 10.1038/s41467-019-13456-z (PMC6923358; doi:10.1038/s41467-019-13456-z)
Supplement: Supplementary file 3 — Reporting Summary [file 41467_2019_13456_MOESM3_ESM.pdf]

nature research

Corresponding author(s): Julien C. Marie

Last updated by author(s): 2019/10/28

Reporting Summary

Nature Research wishes to improve the reproducibility of the work that we publish. This form provides structure for consistency and transparency in reporting. For further information on Nature Research policies, see Authors & Referees and the Editorial Policy Checklist.

Statistics

For all statistical analyses, confirm that the following items are present in the figure legend, table legend, main text, or Methods section.

n/a	Confirmed	
☐	☒	The exact sample size (*n*) for each experimental group/condition, given as a discrete number and unit of measurement
☐	☒	A statement on whether measurements were taken from distinct samples or whether the same sample was measured repeatedly
☐	☒	The statistical test(s) used AND whether they are one- or two-sided<br>*Only common tests should be described solely by name; describe more complex techniques in the Methods section.*
☒	☐	A description of all covariates tested
☒	☐	A description of any assumptions or corrections, such as tests of normality and adjustment for multiple comparisons
☐	☒	A full description of the statistical parameters including central tendency (e.g. means) or other basic estimates (e.g. regression coefficient) AND variation (e.g. standard deviation) or associated estimates of uncertainty (e.g. confidence intervals)
☐	☒	For null hypothesis testing, the test statistic (e.g. *F*, *t*, *r*) with confidence intervals, effect sizes, degrees of freedom and *P* value noted<br>*Give P values as exact values whenever suitable.*
☒	☐	For Bayesian analysis, information on the choice of priors and Markov chain Monte Carlo settings
☒	☐	For hierarchical and complex designs, identification of the appropriate level for tests and full reporting of outcomes
☒	☐	Estimates of effect sizes (e.g. Cohen's *d*, Pearson's *r*), indicating how they were calculated

Our web collection on statistics for biologists contains articles on many of the points above.

Software and code

Policy information about availability of computer code

Data collection

Provide a description of all commercial, open source and custom code used to collect the data in this study, specifying the version used OR state that no software was used.

Data analysis

Provide a description of all commercial, open source and custom code used to analyse the data in this study, specifying the version used OR state that no software was used.

For manuscripts utilizing custom algorithms or software that are central to the research but not yet described in published literature, software must be made available to editors/reviewers. We strongly encourage code deposition in a community repository (e.g. GitHub). See the Nature Research guidelines for submitting code & software for further information.

Data

Policy information about availability of data

All manuscripts must include a data availability statement. This statement should provide the following information, where applicable:

- Accession codes, unique identifiers, or web links for publicly available datasets
- A list of figures that have associated raw data
- A description of any restrictions on data availability

Provide your data availability statement here.

Field-specific reporting

Please select the one below that is the best fit for your research. If you are not sure, read the appropriate sections before making your selection.

☒ Life sciences ☐ Behavioural & social sciences ☐ Ecological, evolutionary & environmental sciences

Life sciences study design

All studies must disclose on these points even when the disclosure is negative.

Sample size	No formal power calculation were performed. The size of each group were robsuta a, data was routinely collected across there independent replicate for each assay and at least three mice per groups
Data exclusions	no data was excluded from the analyses
Replication	All experiments were repeated three times with complet independent set of mice
Randomization	No formal randomization was done as we compared mice with diffrent geneotype and not mice with the same genotype
Blinding	Investigators were blinding to the genotype of the mice, during sample preparation and data analyses

Reporting for specific materials, systems and methods

We require information from authors about some types of materials, experimental systems and methods used in many studies. Here, indicate whether each material, system or method listed is relevant to your study. If you are not sure if a list item applies to your research, read the appropriate section before selecting a response.

Materials & experimental systems

n/a	Involved in the study
☐	☒ Antibodies
☐	☐ Eukaryotic cell lines
☐	☐ Palaeontology
☐	☒ Animals and other organisms
☐	☐ Human research participants
☐	☐ Clinical data

Methods

n/a	Involved in the study
☐	☐ ChIP-seq
☐	☒ Flow cytometry
☐	☐ MRI-based neuroimaging

Antibodies

Antibodies used	CD3 (145-2C11; BD biosciences), CD4 (RM4-5; Biolegend), CD5 (53-7.3; BD biosciences), CD8a (53.6.7; BD biosciences), CD24 (M1/69; BD biosciences), CD40L (MR1; eBioscience), CD45.1 (A20; BD biosciences), CD45.2 (104; BD biosciences), CD45 (30-F11; BD biosciences), CD69 (H1.2F3; BD biosciences), CD80 (16-10A1 eBioscience) CD90.2 (53-2.1; BD bioscience), CD90.1 (Ox7 BD biosciences), CCR7 (4B12; BD biosciences), CXCR3 (CXCR3-173; eBioscience), EpCAM (G8.8; eBioscience), H2Kb (AF6-88-5.5.3 eBioscience) LY51 (6C3/BP-1; BD biosciences), PD1 (EH12.2H7; BioLegend), S1P1 (T4-H28; R&D systems), Qa2 (1-1-2 BD biosciences) TCRB; (BD biosciences). F RANKL (IK22-5; BD biosciences), IFN-g (XMG1.2 BD bioscience) TNF-a (MP6XT22 BD biosciences) activated caspase 3 (C92-605; BD biosciences), and BIM (C34C5; Cell Signaling Technology) AIRE (5H12; eBioscience), Foxp3 (FJK-16S; eBioscience), Helios (22F6; BioLegend), Ki67 (B56; BD biosciences), and T-Bet (eBio4B10; eBioscience) Anti-rat MTS10 (Pharmingen), rabbit anti-K14 (AF64, Covance Research), Alexa Fluor 488 anti-AIRE (5H12, ebioscience), Alexa Fluor 647-conjugated anti-Helios (22F6, eBioscience), and AlexaFluor 488-conjugated anti-Foxp3 (eBioscience) .
Validation	All the antibodies are against mice and have been routinely used by numerous other publications. The antibodies used have been validated by the manufacters reporting their own tests and publications that used them. An adequate titration was made for each antibody prior further experiments

Eukaryotic cell lines

Policy information about cell lines

Cell line source(s)	*State the source of each cell line used.*
Authentication	*Describe the authentication procedures for each cell line used OR declare that none of the cell lines used were authenticated.*
Mycoplasma contamination	*Confirm that all cell lines tested negative for mycoplasma contamination OR describe the results of the testing for mycoplasma contamination OR declare that the cell lines were not tested for mycoplasma contamination.*
Commonly misidentified lines (See ICLAC register)	*Name any commonly misidentified cell lines used in the study and provide a rationale for their use.*

Palaeontology

Specimen provenance	*Provide provenance information for specimens and describe permits that were obtained for the work (including the name of the issuing authority, the date of issue, and any identifying information).*
Specimen deposition	*Indicate where the specimens have been deposited to permit free access by other researchers.*
Dating methods	*If new dates are provided, describe how they were obtained (e.g. collection, storage, sample pretreatment and measurement), where they were obtained (i.e. lab name), the calibration program and the protocol for quality assurance OR state that no new dates are provided.*

☐ Tick this box to confirm that the raw and calibrated dates are available in the paper or in Supplementary Information.

Animals and other organisms

Policy information about studies involving animals; ARRIVE guidelines recommended for reporting animal research

Laboratory animals	Mus musculus, CD4-Cre;Tgfbr2fl/fl, CD4-Cre;Smad4fl/fl mice, CD4-Cre;tif1gfl/fl mice and , CD4-Cre; tif1gfl/fl; Smad4fl/fl (C57BL/6 background) Foxp3GFP . AIRE-KO mice Rag2-KO deficient mice (Rag-KO), CD3-KO , Thy1.1 (CD90.1), CD45.1, mice. male and female were used. Except of Rag2-KO, CD3KO used at 6-8 week of age,and for FTOC (embrio E17.5) the other mouse strains were used between 7-10 daysof age
Wild animals	The study did not involved wild animals
Field-collected samples	The study did not involved field-collected samples
Ethics oversight	Mice were housed and bred in a specific pathogen free animal facility, AniCan, at the C R C L. Experiments were performed in accordance with the animal care guidelines of the European Union and French laws and were validated by the local Animal Ethic Evaluation Committee (CECCAPP).

Note that full information on the approval of the study protocol must also be provided in the manuscript.

Human research participants

Policy information about studies involving human research participants

Population characteristics	*Describe the covariate-relevant population characteristics of the human research participants (e.g. age, gender, genotypic information, past and current diagnosis and treatment categories). If you filled out the behavioural & social sciences study design questions and have nothing to add here, write "See above."*
Recruitment	*Describe how participants were recruited. Outline any potential self-selection bias or other biases that may be present and how these are likely to impact results.*
Ethics oversight	*Identify the organization(s) that approved the study protocol.*

Note that full information on the approval of the study protocol must also be provided in the manuscript.

Clinical data

Policy information about clinical studies

All manuscripts should comply with the ICMJE guidelines for publication of clinical research and a completed CONSORT checklist must be included with all submissions.

Clinical trial registration	*Provide the trial registration number from ClinicalTrials.gov or an equivalent agency.*
Study protocol	*Note where the full trial protocol can be accessed OR if not available, explain why.*
Data collection	*Describe the settings and locales of data collection, noting the time periods of recruitment and data collection.*
Outcomes	*Describe how you pre-defined primary and secondary outcome measures and how you assessed these measures.*

ChIP-seq

Data deposition

☐ Confirm that both raw and final processed data have been deposited in a public database such as GEO.

☐ Confirm that you have deposited or provided access to graph files (e.g. BED files) for the called peaks.

Data access links<br>*May remain private before publication.*	*For "Initial submission" or "Revised version" documents, provide reviewer access links. For your "Final submission" document, provide a link to the deposited data.*

Files in database submission

Provide a list of all files available in the database submission.

Genome browser session (e.g. UCSC)

Provide a link to an anonymized genome browser session for "Initial submission" and "Revised version" documents only, to enable peer review. Write "no longer applicable" for "Final submission" documents.

Methodology

Replicates

Describe the experimental replicates, specifying number, type and replicate agreement.

Sequencing depth

Describe the sequencing depth for each experiment, providing the total number of reads, uniquely mapped reads, length of reads and whether they were paired- or single-end.

Antibodies

Describe the antibodies used for the ChIP-seq experiments; as applicable, provide supplier name, catalog number, clone name, and lot number.

Peak calling parameters

Specify the command line program and parameters used for read mapping and peak calling, including the ChIP, control and index files used.

Data quality

Describe the methods used to ensure data quality in full detail, including how many peaks are at FDR 5% and above 5-fold enrichment.

Software

Describe the software used to collect and analyze the ChIP-seq data. For custom code that has been deposited into a community repository, provide accession details.

Flow Cytometry

Plots

Confirm that:

☐ The axis labels state the marker and fluorochrome used (e.g. CD4-FITC).

☒ The axis scales are clearly visible. Include numbers along axes only for bottom left plot of group (a 'group' is an analysis of identical markers).

☒ All plots are contour plots with outliers or pseudocolor plots.

☒ A numerical value for number of cells or percentage (with statistics) is provided.

Methodology

Sample preparation

Thymus and neck draining lymp nodes were harvested and mechanically minced using glass slides in completed RPMI 5% FCS (Life technologies). Ears and colon were collected. Colon were flushed with cold PBS(Life Technologies). For lamina propri large intestines were removed of fat longitudinally opened and washed in PBS. Intestines and ears were cut into small pieces with sclapel/To eliminate epithelial cell colon was incubated with 5mM EDTA, 1mM DTT (Sigma Aldrich) at 37°C. Epithelial cells and IEL were then separated from tissue. Ear and colon were then digested in RPMI medium (Life Technologies) containing 20% FCS (Life Technologies), DNAse I (Roche) at 100µg/ml and Collagenase from Clostridium Histolyticum (Sigma Aldrich) at 0.6mg/ml. Intestinal LP were harvested from a 40% percoll (fischer scientic) gradient run for 20min at 1300 G.
For mTEC isolation: thymus were minced with scapel Fand digested in PBS containing 1 mg/ml collagenase D (Sigma Aldrich) and 2 µg/ml DNaseI (Roche). Remaining hematopoietic cells of the preparation were removed with anti-CD45 magnetic beads (Miltenyi Biotec).
Surface staining was performed in PBS (Life technology) 5% FCS (Life technology), PBS1X Life technologies, Azide 0.02% (Sigma).
For cytokine production analyses cells were incubated in completed RPMI 5% FCS, 5%HEPES with 500 ng/ml PMA (Sigma Aldrich) and 500 ng/ml ionomycin (Sigma Aldrich) in presence of Brefeldin A (1/1000) (Invitrogen) for 3 hours à 37°C 5% CO2.
For intracellular stainings (Cytokines, BIM) we used cytofix cytoperm kit (BD) according to the manufacturer's protocol.
For intranuclear staining (Helios, T-BET, AIRE K67) we used intra nuclear staining kit according manufacturer's instructions (eBioscience).

Instrument

LSR Fortesssa (BD)
ARIA II Sorter

Software

Flow Joe 10

Cell population abundance

Facs ARIA II sort purity was >95% confirmed by analysis on a second cytometer before cells were used for further experiments

Gating strategy

Gating strategies including sort strategies are depicted for each experiment in the article. For FSC/SSC population were gated as within lymphocyte gates or epithelial gate as has been traditionally done. Singlets were gated on FSC-A/FSC-W as well as SSC-A/SSC-W

☒ Tick this box to confirm that a figure exemplifying the gating strategy is provided in the Supplementary Information.

Magnetic resonance imaging

Experimental design

Design type

Indicate task or resting state; event-related or block design.

Design specifications

Specify the number of blocks, trials or experimental units per session and/or subject, and specify the length of each trial or block (if trials are blocked) and interval between trials.

Behavioral performance measures

State number and/or type of variables recorded (e.g. correct button press, response time) and what statistics were used to establish that the subjects were performing the task as expected (e.g. mean, range, and/or standard deviation across subjects).

Acquisition

Imaging type(s)

Specify: functional, structural, diffusion, perfusion.

Field strength

Specify in Tesla

Sequence & imaging parameters

Specify the pulse sequence type (gradient echo, spin echo, etc.), imaging type (EPI, spiral, etc.), field of view, matrix size, slice thickness, orientation and TE/TR/flip angle.

Area of acquisition

State whether a whole brain scan was used OR define the area of acquisition, describing how the region was determined.

Diffusion MRI ☐ Used ☐ Not used

Preprocessing

Preprocessing software

Provide detail on software version and revision number and on specific parameters (model/functions, brain extraction, segmentation, smoothing kernel size, etc.).

Normalization

If data were normalized/standardized, describe the approach(es): specify linear or non-linear and define image types used for transformation OR indicate that data were not normalized and explain rationale for lack of normalization.

Normalization template

Describe the template used for normalization/transformation, specifying subject space or group standardized space (e.g. original Talairach, MNI305, ICBM152) OR indicate that the data were not normalized.

Noise and artifact removal

Describe your procedure(s) for artifact and structured noise removal, specifying motion parameters, tissue signals and physiological signals (heart rate, respiration).

Volume censoring

Define your software and/or method and criteria for volume censoring, and state the extent of such censoring.

Statistical modeling & inference

Model type and settings

Specify type (mass univariate, multivariate, RSA, predictive, etc.) and describe essential details of the model at the first and second levels (e.g. fixed, random or mixed effects; drift or auto-correlation).

Effect(s) tested

Define precise effect in terms of the task or stimulus conditions instead of psychological concepts and indicate whether ANOVA or factorial designs were used.

Specify type of analysis: ☐ Whole brain ☐ ROI-based ☐ Both

Statistic type for inference
(See Eklund et al. 2016)

Specify voxel-wise or cluster-wise and report all relevant parameters for cluster-wise methods.

Correction

Describe the type of correction and how it is obtained for multiple comparisons (e.g. FWE, FDR, permutation or Monte Carlo).

Models & analysis

n/a	Involved in the study
☐	☐ Functional and/or effective connectivity
☐	☐ Graph analysis
☐	☐ Multivariate modeling or predictive analysis

Functional and/or effective connectivity

Report the measures of dependence used and the model details (e.g. Pearson correlation, partial correlation, mutual information).

Graph analysis

Report the dependent variable and connectivity measure, specifying weighted graph or binarized graph, subject- or group-level, and the global and/or node summaries used (e.g. clustering coefficient, efficiency, etc.).

Multivariate modeling and predictive analysis

Specify independent variables, features extraction and dimension reduction, model, training and evaluation metrics.
